# Comparison of the levels of neopterin, CRP, and IL-6 in patients infected with and without SARS-CoV-2

**DOI:** 10.1016/j.heliyon.2022.e09371

**Published:** 2022-05-04

**Authors:** Satoshi Hara, Tama Sanatani, Natsuo Tachikawa, Yukihiro Yoshimura, Nobuyuki Miyata, Hiroaki Sasaki, Risa Kuroda, Chinatsu Kamikokuryo, Tomohiro Eguchi, Shuhei Niiyama, Yasuyuki Kakihana, Hiroshi Ichinose

**Affiliations:** a School of Life Science and Technology, Tokyo Institute of Technology, Yokohama, Japan; b Division of Infectious Disease, Yokohama Municipal Citizen's Hospital, Yokohama, Kanagawa, Japan; c Asano Hospital, Yokohama, Kanagawa, Japan; d Yokohama Pain Clinic, Yokohama, Kanagawa, Japan; e Department of Emergency and Intensive Care Medicine, Kagoshima University Graduate School of Medical and Dental Sciences, Sakuragaoka, Kagoshima, Japan

**Keywords:** COVID-19, SARS-CoV-2, Neopterin, CRP, IL-6, Viral infection, Biomarker

## Abstract

**Background:**

Neopterin (NP) is a biomarker for activated cellular immunity and is elevated in diseases including viral and bacterial infections, autoimmune diseases, and cancer. However, the clinical assessment of neopterin has not been used for these disorders because the physiological significance of measuring NP is obscure. It would be important to compare the NP profiles with those of other inflammation markers especially in relatively early phase of patients to reveal the significance of NP measurements in pathological states.

**Methods:**

Plasma NP, biopterin, CRP, and IL-6 levels were measured in 46 patients with Coronavirus Disease 2019 (COVID-19) and 23 patients with non-COVID-19 disorders. The correlations between these markers were analyzed in the COVID-19 and non-COVID-19 patients independently.

**Results:**

The NP levels were significantly higher in the COVID-19 patients than in the non-COVID-19 patients, while biopterin, CRP and IL-6 were not changed significantly. The NP levels were found to show a weak negative correlation against the days after onset in the COVID-19 patients (*rs* = –0.348, *p* = 0.0192), suggesting that the elevation of NP would be an early event of viral infection. Correlations between NP and CRP, or between NP and IL-6 in COVID-19 patients were weaker than that between CRP and IL-6.

**Conclusions:**

The elevation of NP levels was supposed to be distinct from those of CRP and IL-6 in relatively early and mild COVID-19 patients. Our data suggest that NP is produced at the early phase of infection by different signaling pathways and/or cells from those of CRP and IL-6. Further study on the signaling pathway to induce NP is expected.

## 1 Introduction

Neopterin (NP) has been established as a biomarker for the activation of cellular immunity [1]. NP and biopterin (BP) are unconjugated pteridine compounds and are synthesized from GTP through several enzymatic reactions. GTP cyclohydrolase I (GCH) is the first and rate-limiting enzyme. A bioactive form of BP, 5,6,7,8-6*R*-L-*erythro*-tetrahydrobiopterin, is an essential cofactor for the syntheses of monoamines and nitric oxide and for lipid metabolism [2]. Despite these well-known functions of BP, the physiological meaning of NP has not yet been clarified.

Coronavirus Disease 2019 (COVID-19), caused by severe acute respiratory syndrome coronavirus 2 (SARS-CoV-2) infection, has rapidly spread across the world. To date, 216 million people have been infected, resulting in 4.5 million deaths [3]. The production of proinflammatory cytokines through activation of the immune system by SARS-CoV-2 causes a cytokine storm, although the production of cytokines is necessary for an antiviral immune response [4]. The cytokine storm, including an overproduction of interleukin-6 (IL-6), is thought to cause acute respiratory distress syndrome (ARDS). In fact, IL-6 and the inflammatory marker C-reactive protein (CRP) have been reported to correlate with COVID-19 symptoms [5, 6].

NP is thought to be synthesized by monocytes/macrophages stimulated by interferon gamma (IFN-γ) [1]. NP levels in the plasma increase in various pathological conditions, including infections, autoimmune diseases and cancer. Infection with a variety of viruses, i.e., hepatitis, cytomegalovirus and dengue virus were reported to increase NP levels in the plasma [7, 8, 9]. In COVID-19 patients, an increase in NP in serum, cerebrospinal fluid and feces and an association with the severity of symptoms have been reported [10, 11, 12, 13, 14, 15, 16]. Although these reports clearly suggest the relevance of serum NP as a biomarker of viral infection, NP levels have not been utilized clinically, probably due to the lack of physiological meaning of NP, including information about the cells and signaling pathways that produce NP under pathological conditions.

In the present study, we measured the levels of NP, CRP, and IL-6 in patients suffering from COVID-19 and those with noninfectious respiratory symptoms and compared their correlations to examine whether these values would reflect similar biological processes. We analyzed 69 patients, including 23 non-COVID-19 patients, who were hospitalized in infectious disease internal medicine. Our data suggest that NP synthesis is upregulated at the early phase of infection by a distinct pathway and/or cells from those of CRP and IL-6 in patients with COVID-19.

## 2 Methods

### 2.1 Study population

We analyzed plasma of 69 hospitalized patients between April 2020 and February 2021 in Japan. Infection with SARS-CoV-2 was confirmed by reverse transcriptase-PCR. Plasma samples were stored in a deep freezer until use. In this study, the severity of symptoms was defined as follows: mild (no pneumonia), moderate (pneumonia without oxygen inhalation), severe (pneumonia required for oxygen inhalation) and critical (ICU admission or required for mechanical ventilation). Pneumonia was diagnosed by CT scan. All patients were unvaccinated for COVID-19. This study was approved by the ethics committees of the Tokyo Institute of Technology, Kagoshima University and Yokohama Municipal Citizen's Hospital.

### 2.2 Measurements of plasma NP and BP

Because the reduced forms of pteridines, tetrahydrobiopterin, dihydrobiopterin and dihydroneopterin (H_2_NP), are labile, the plasma samples were oxidized with a one-tenth volume of iodine solution (1% iodine/2% KI) in the presence of 0.4 M perchloric acid to convert their oxidized forms, BP and NP, respectively [17]. The excess iodine was then eliminated with the addition of ascorbic acid and followed by centrifugation for deproteinization. The concentrations of NP and BP were determined by an HPLC-fluorescence detection system. The pteridines were separated using a Shim-pack Velox Biphenyl column (2.7 μm, 2.1 mm × 100 mm, Shimadzu, Japan) with 10 mM Na-phosphate (pH 6.9) as a mobile phase. Values were presented as mean ± SEM. A normal level of NP was set at 10 pmol/ml [18].

### 2.3 Measurements of plasma CRP and IL-6

Plasma CRP and IL-6 concentrations were determined by using a CRP ELISA Kit (Immunology Consultants Laboratory, USA) and Human IL-6 Quantikine ELISA Kit (R&D Systems, USA), respectively. CRP and IL-6 in one PCR-positive patient with severe symptoms, and CRP in one PCR-negative with moderate symptoms could not be measured because of a shortage of the sample. Values were presented as mean ± SEM. Normal levels of CRP and IL-6 were set as 0.3 mg/dl and 8 pg/ml, respectively.

### 2.4 Stability of H_2_NP

Dihydroneopterin triphosphate was prepared by the enzymatic reaction of *E. coli* GTP cyclohydrolase I as previously described [19]. The triphosphate-moiety was eliminated by the incubation of 50 μM dihydroneopterin triphosphate with 1 U of alkaline phosphatase (Roche, Switzerland) in 50 mM Tris-HCl (pH 7.4) and 1 mM MgCl_2_ for 50 min at 37 °C. Alkaline phosphatase was removed with Vivaspin 500 (Molecular weight cutoff 3k, Sartorius, Germany), and flow-through was stored at –80 °C. For spiking experiments, we added one-fourth volume of 100 nM H_2_NP to the plasma sample, and immediately deproteinized with 0.2 M perchloric acid and 0.1 mM EDTA following centrifugation. NP and H_2_NP in the supernatant were separated and measured using HPLC with a post-column oxidation method [20].

### 2.5 Statistical analyses

All statistical analyses were performed with EZR software [21]. Categorical data were analyzed by using Fisher's exact test. For comparison of continuous data among the groups, the Mann–Whitney *U* test and Kruskal–Wallis test with Steel-Dwass post hoc test were used. Correlations were measured with Spearman correlation. *P* values <0.05 were regarded as statistically significant.

## 3 Results

The population in this study comprised 46 PCR-positive COVID-19 patients, of whom 7 had mild symptoms, 15 had moderate symptoms, 21 had severe symptoms and 3 had critical symptoms, and 23 PCR-negative non-COVID-19 patients who showed 12 mild symptoms, 8 moderate symptoms, 2 severe symptoms and 1 critical symptom. The mean ages and the distribution of sex between COVID-19 and non-COVID-19 patients were not significantly different (Table 1). The diseases of non-COVID-19 patients were 11 cases of pneumonia and 12 cases of other diseases with fever, diarrhea, dysgeusia, infectious mononucleosis, or viral meningitis.

**Table 1:** Demographic data in this study.

	COVID-19 (n = 46)	Non-COVID-19 (n = 23)	*p*-value
Symptoms			
Mild	7	12	
Moderate	15	8	
Severe	21	2	
Critical	3	1	
Sex, F/M	18/28	8/15	0.796^a^
Age (mean ± SEM)	56.2 ± 3.2	65.3 ± 4.2	0.0904^b^

a The statistical analysis was performed with Fisher's exact test.

b The statistical analysis was performed with Mann-Whitney *U*-test.

**Figure 1:**
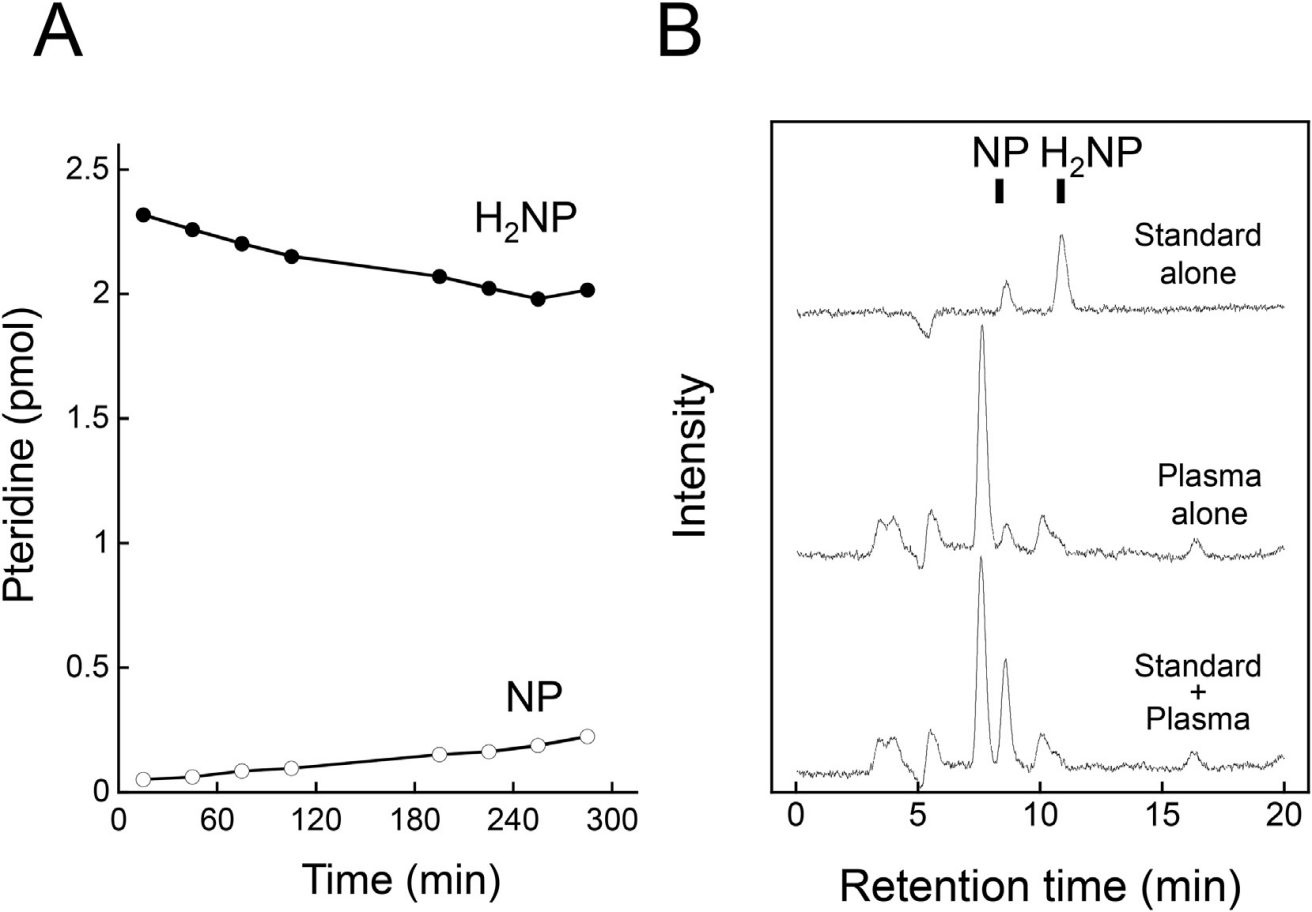
Conversion of H_2_NP to NP in PBS (A) and in human plasma (B). (A) H_2_NP in PBS was place on the autosampler at 10 °C in the dark, and then analyzed by HPLC with post-column oxidation method at times indicated. (B) Exogenous H_2_NP was added to plasma. After deproteinization, samples were analyzed. PBS was added instead of H_2_NP for the preparation of the plasma alone sample.

We measured the NP and BP levels after oxidation with iodine to convert reduced forms of pteridines, i.e. BH4, BH2, and H_2_NP, to their fully oxidized forms. We examined the stability of H_2_NP at neutral pH. We found that H_2_NP was gradually converted to NP (Figure 1A). Indeed, when exogenous H_2_NP was added to a plasma sample, the content of NP was elevated (Figure 1B). These results suggest that the sum of reduced and oxidized forms of pteridines might reflect total alterations in the biosynthesis of NP and BP better than the amount of an oxidized form alone.

**Figure 2:**
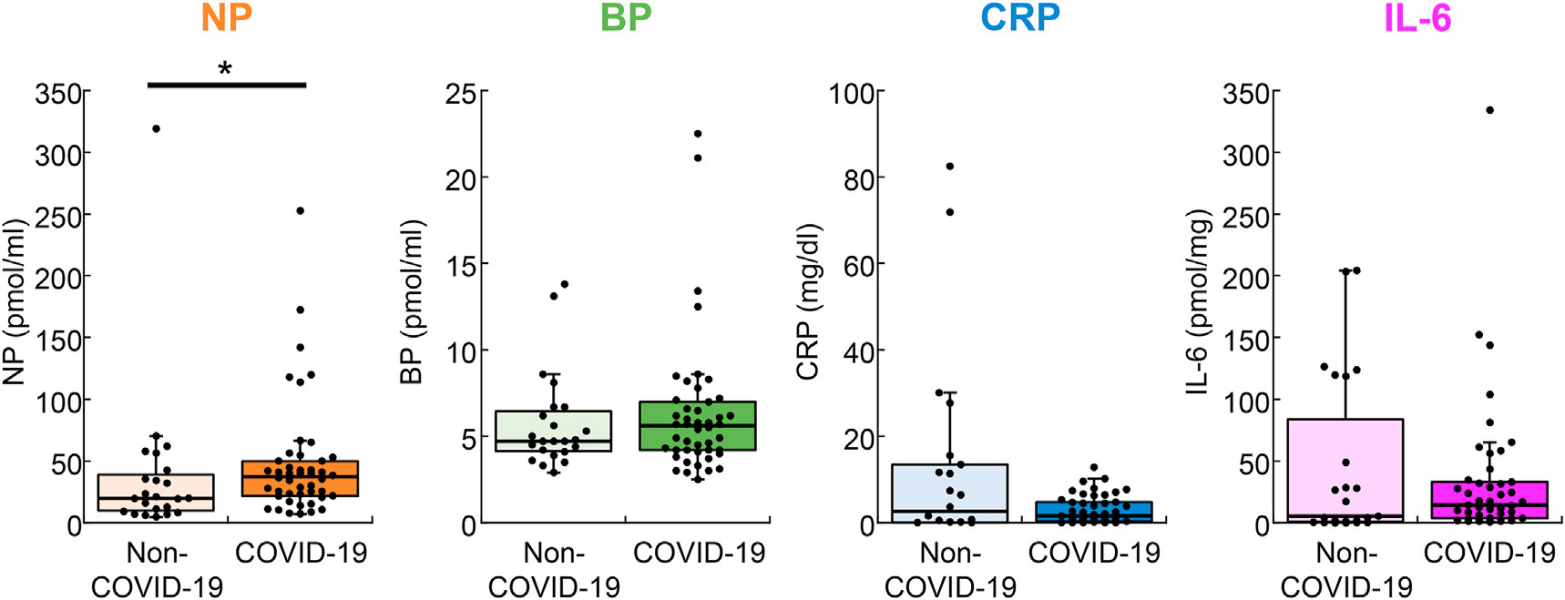
Concentrations of plasma NP, BP, CRP and IL-6 in COVID-19 (n = 46 for NP and BP, and n = 45 for CRP and IL-6) and non-COVID-19 patients (n = 23 in non-COVID-19 patients for NP, BP and IL-6 and 22 for CRP). ∗∗*p* < 0.01, Mann–Whitney *U* test. The boxes represent the median and interquartile ranges. Whiskers represent minimum and maximum 1.5 interquartile range and dots are outliers.

To identify the association of the levels of NP, BP, CRP and IL-6 with SARS-CoV-2 infection, we first compared them between patients infected with and without SARS-CoV-2 (Figure 2). The mean level of NP in COVID-19 patients was significantly higher than that in non-COVID-19 patients (47.9 ± 6.9 vs. 38.8 ± 13.4, *p* = 0.0417). On the other hand, BP (6.3 ± 0.6 vs. 5.8 ± 0.6, *p* = 0.628) and the well-known inflammatory markers CRP (3.1 ± 0.5 vs. 13.0 ± 4.8, *p* = 0.593) and IL-6 (34.3 ± 8.5 vs. 46.3 ± 13.9, *p* = 0.448) did not differ significantly between the COVID-19 and non-COVID-19 groups. To evaluate how accurately the infection was differentiated, ROC curves were generated between COVID-19 and non-COVID-19 patients (data not shown). The obtained AUC values of NP, BP, CRP and IL-6 were 0.652 (95% CI, 0.502–0.801), 0.536 (95% CI, 0.391–0.681), 0.541 (95% CI, 0.359–0.723) and 0.557 (95% CI, 0.388–0.726), respectively.

**Figure 3:**
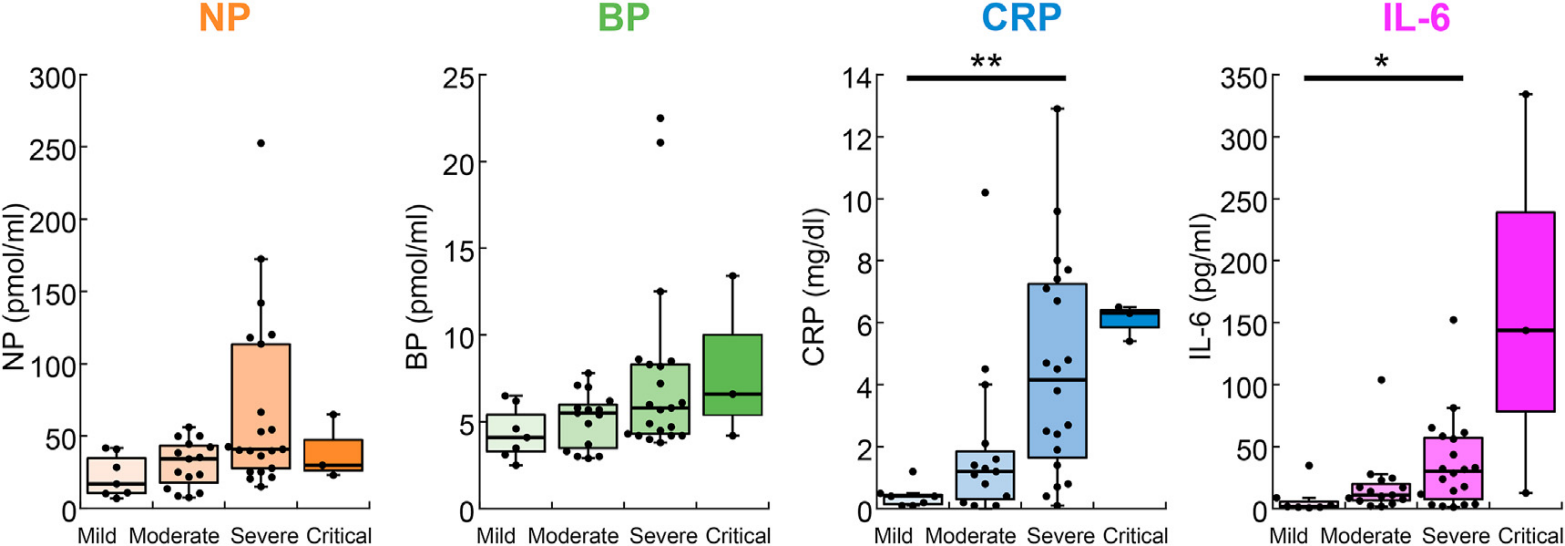
Concentrations of plasma NP, BP, CRP and IL-6 in COVID-19 patients with mild (n = 7), moderate (n = 15), severe (n = 21 for NP and BP, and n = 20 for CRP and IL-6) and critical (n = 3) symptoms. ∗*p* < 0.05, ∗∗*p* < 0.01, Kruskal–Wallis test with Steel-Dwass post hoc test. The boxes represent the median and interquartile ranges. Whiskers represent minimum and maximum 1.5 interquartile range and dots are outliers.

We next tested the association of the biomarkers with the severity of symptoms in COVID-19 patients (Figure 3). We found significant increases in severe patients compared with mild for CRP (4.5 ± 0.8 vs. 0.41 ± 0.1, *p* = 0.0060) and IL-6 (36.2 ± 8.1 vs. 7.3 ± 4.4, *p* = 0.040). No significant difference was observed in NP and BP, although they tended to increase with the severity of symptoms. Collectively, all biomarkers seem to be elevated with the severity of symptoms, while CRP was the most sensitive indicator.

**Figure 4:**
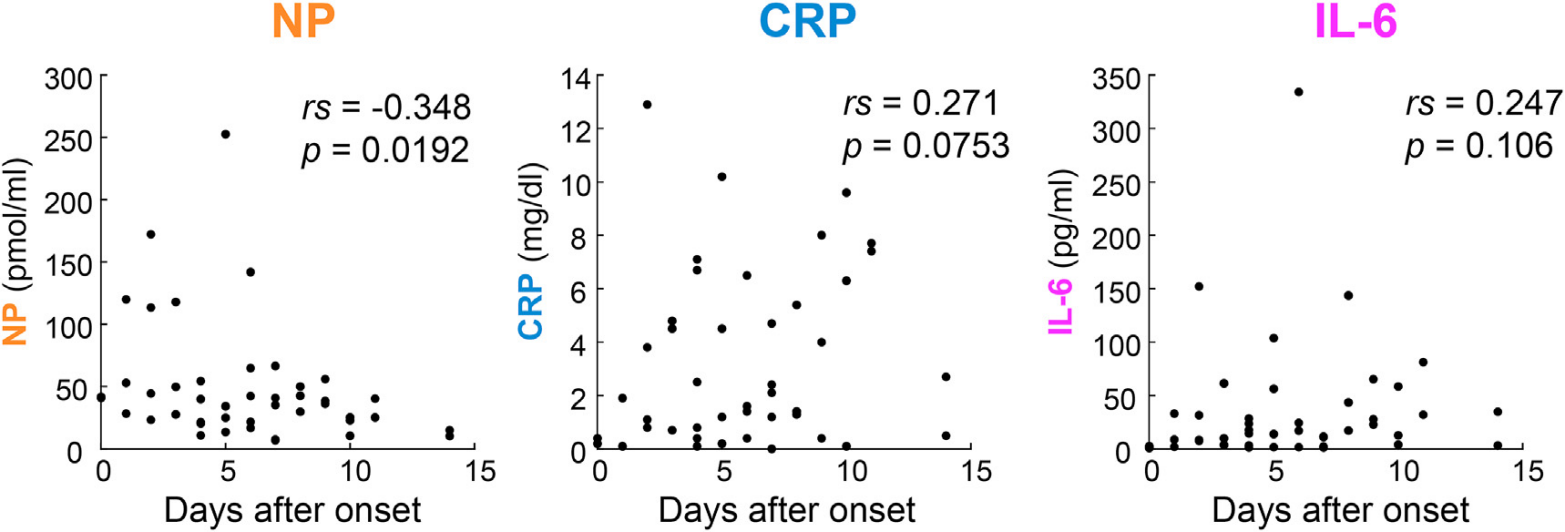
Concentrations of NP, CRP and IL-6 in COVID-19 patients after the onset of symptoms expressed as days. n = 46, 45 and 45, respectively. Spearman's correlation coefficients (*rs*) and *p*-values are shown in the plots.

The difference in NP between COVID-19 and non-COVID-19 patients was significant, even though there was no significant increase with severity, suggesting that NP was elevated at early or mild stage of the symptoms. Thus, we plotted the levels of NP, CRP, and IL-6 against the days after onset in the COVID-19 patients (Figure 4). NP was found to show a weak negative correlation (*rs* = –0.348, *p* = 0.0192). In contrast, CPR and IL-6 showed almost no correlation. This suggests that NP increased at an early stage of infection and decreased over the course of this study. Notably, the NP values of two patients with mild symptoms on Day 0 were 41.8 and 40.9 pmol/ml, which were clearly higher than the normal range of NP, while their CRP and IL-6 levels were within normal ranges.

**Figure 5:**
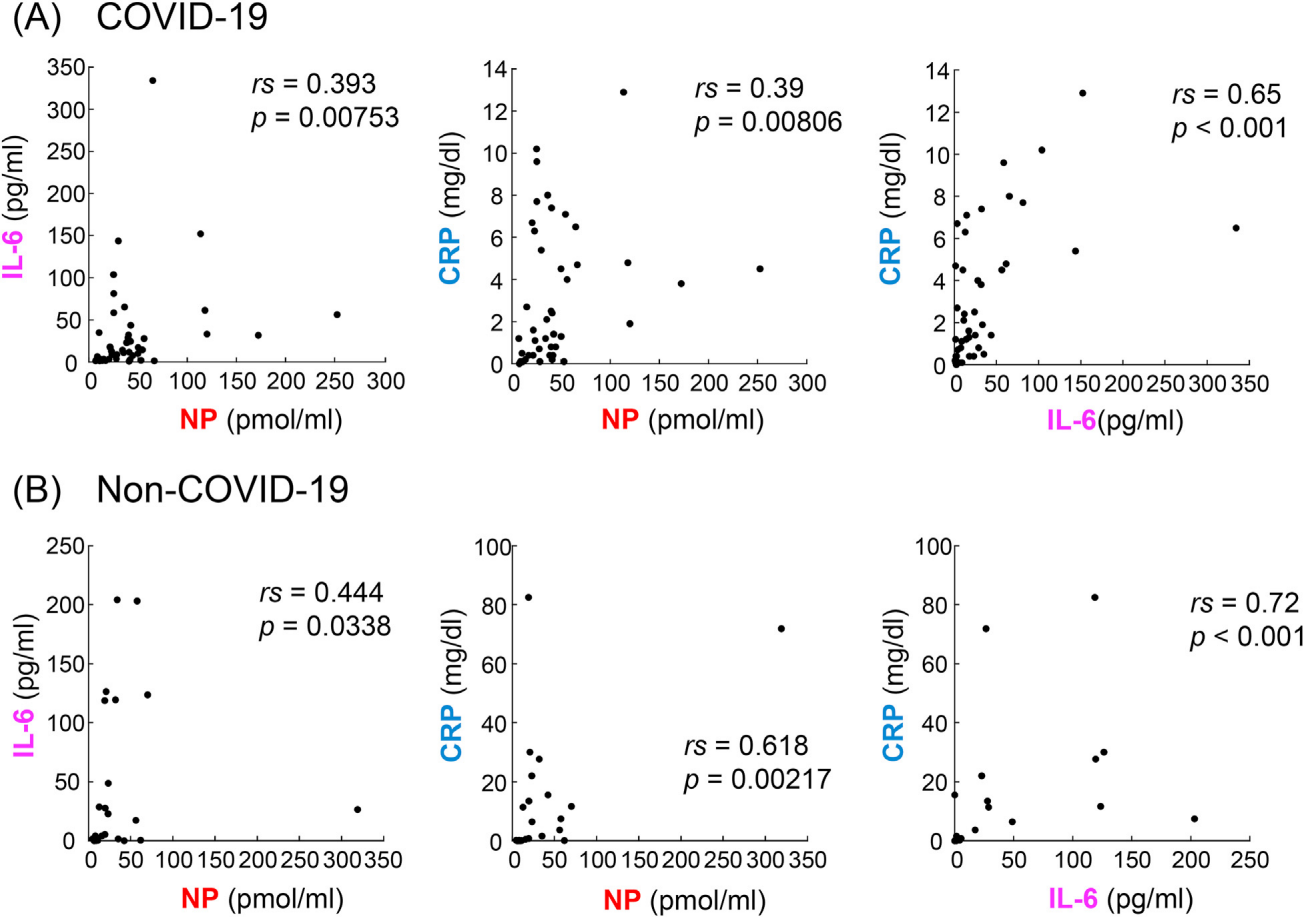
Correlations among NP, CRP and IL-6 in COVID-19 (A) and non-COVID-19 (B) patients. n = 45 in each panel in COVID-19 patients. n = 23 for IL-6-NP and n = 22 for CRP-NP and CRP-IL-6 in non-COVID-19 patients. Spearman's correlation coefficients (*rs*) and *p*-values are shown in the plots.

To examine whether NP, CRP, and IL-6 were produced through the same signaling pathway in viral infection (COVID-19 patients) and other causes (non-COVID-19 patients), we analyzed their correlations (Figure 5). We found that CRP correlated with IL-6 in either COVID-19 or non-COVID-19 patients (*rs* = 0.65, *p* < 0.001 and *rs* = 0.72, *p* < 0.001, respectively), and that NP correlated with CRP in non-COVID-19 patients (*rs* = 0.618, *p* = 0.00217). In contrast, a weak correlation between NP and CRP was observed in COVID-19 patients (*rs* = 0.39, *p* = 0.00806). Correlations of NP with IL-6 in both COVID-19 and non-COVID-19 patients were also weaker (*rs* = 0.393, *p* = 0.00753 and *rs* = 0.444, *p* = 0.0338). These data suggest that the signaling pathway and mechanisms inducing the plasma levels of NP should be different from those of CRP and IL-6 in COVID-19 at least partly.

## 4 Discussion

In the present study, we measured not only NP but also CRP and IL-6, which have been correlated with SARS-CoV-2 infection and the severity of symptoms [5, 6] in the same patients, and analyzed the relationship among them. The comparisons between COVID-19 and non-COVID-19 patients showed that only NP could differentiate SARS-CoV-2 infection from non-COVID-19 patients in this study (Figure 1). Greater elevation of NP levels in patients with viral infection was also reported in other viral diseases [8, 9, 18, 22]. Rainer *et al.* [22] proposed that the ratios of CRP to NP can be used to distinguish viral and bacterial infections. Collectively, NP in plasma should be a more sensitive biomarker for viral infection, including SARS-CoV-2, than CRP and IL-6.

Since NP was elevated significantly with SARS-CoV-2 infection but not with severity (Figures 2 and 3), it is suggested that early and mild symptoms were enough to increase plasma NP levels. The NP levels showed a weak negative correlation with the days after onset, while CRP and IL-6 did not show significant alterations (Figure 4). The increases in NP might occur earlier than the appearance of symptoms. Notably, two patients on Day 0 showed higher NP levels with normal CRP and IL-6 values. Zheng *et al.* reported early increases in NP compared with those of CRP in SARS-CoV patients [18]. Taken together, NP may increase in the very early stages of viral infection and could be an early diagnostic marker to distinguish viral infection from other causes of diseases.

The different diagnostic characteristics mentioned above also suggested that NP should be synthesized through different signaling pathways and cells than those of CRP and IL-6, especially in cases of early viral infection. In Figure 5, we analyzed the correlations among NP, CRP, and IL-6 in COVID-19 and non-COVID-19 patients separately. Compared with moderate correlation between CRP and IL-6 observed in COVID-19 patients, the correlations of NP levels with either CRP or IL-6 were weak. Therefore, it is suggested that NP may be increased independent of IL-6 and CRP elevation in cases of viral infection. In contrast, there was a moderate correlation of NP with CRP in non-COVID-19 but not in COVID-19 (Figure 5). However, there is a limitation in interpretation of the correlation analysis in non-COVID-19 patients, because two non-COVID-19 patients showed extremely high CRP levels (71.9 and 82.5 mg/dl) affecting the correlation analysis.

In the present study, we measured NP after iodine oxidation of the plasma to oxidize H_2_NP to NP, whereas many previous studies analyzed the NP levels without the oxidation step using an ELISA kit against NP. NP in the plasma is derived from dihydroneopterin triphosphate, a product of the GCH reaction, through H_2_NP. We previously reported that there were considerable amounts of H_2_NP in human cerebrospinal fluids [23]. Thus, the measurements of NP alone may be affected by the storage conditions of samples. The measurements of the sum of NP and H_2_NP after iodine oxidation would enhance its sensitivity and reliability to discriminate the samples with higher NP values from normal ones. In addition, iodine oxidation of specimens can reduce the risk of infection during operation due to inactivation of viruses.

NP is thought to be synthesized in monocytes/macrophages activated by IFN-γ. However, it was reported that the NP levels in patients with chronic heart failure were increased without IFN-γ elevation and correlated with TNF-α [24]. Furthermore, patients suffering from Mendelian susceptibility to mycobacterial disease (MSMD) syndrome, who are deeply impaired or defective in the IFN-γ/IL-12/IL-23-dependent pathway, were reported to show increased NP levels [25]. In addition, the cytokine storm found in COVID-19 may be distinct from macrophage activation syndrome [26], while increased IFN-γ levels were reported in COVID-19 patients [27]. Considering these reports and our present results, there would be an IFN-γ-independent pathway for NP production.

The limitation of our study is the rather small number of patients. The study needs to be replicated with a greater number of patients and more extensive measurements of other cytokines to reveal a possible new pathway to induce NP after viral infection.

## 5 Conclusion

In the present study, we compared the inflammation markers NP, CRP, and IL-6 in the plasma of COVID-19 and non-COVID-19 patients. The NP levels significantly distinguished patients infected with SARS-CoV-2 from noninfected patients. Correlation analyses among the three biomarkers showed little correlation of NP with CRP and IL-6 in COVID-19 patients, while CRP levels were strongly correlated with the IL-6 levels. Collectively, we suggest a distinct signaling pathway and/or cells for the induction of NP from those of CRP and IL-6 in patients with COVID-19. The use of NP in a clinical situation would be beneficial for distinguishing early patients with viral infection.

## Declarations

### Author contribution statement

Hiroshi Ichinose: conceived and designed the experiments; analyzed and interpreted the data; wrote the paper.

Satoshi Hara, performed the experiments; analyzed and interpreted the data; wrote the paper.

Tama Sanatani: performed the experiments.

Chinatsu Kamikokuryo; performed the experiments; analyzed and interpreted the data.

Tomohiro Eguchi, Shuhei Niiyama, Yasuyuki Kakihana: analyzed and interpreted the data.

Natsuo Tachikawa, Yukihiro Yoshimura, Nobuyuki Miyata, Hiroaki Sasaki, Risa Kuroda: contributed reagents, materials, analysis tools or data.

### Funding statement

This work was supported by AMED under (Grant Number JP20he0622039), and by a subsidy for Project Support (Trial Assistance) of life science from Kihara Memorial Yokohama Foundation for the Advancement of Life Sciences.

### Data availability statement

Data included in article/supplementary material/referenced in article.

### Declaration of interests statement

The authors declare no conflict of interest.

### Additional information

No additional information is available for this paper.
